# Supplementary material for: Integrating multiple spatial transcriptomics data using community-enhanced graph contrastive learning
Source: PLoS Comput Biol. 2025 Apr 3;21(4):e1012948. doi: 10.1371/journal.pcbi.1012948 (PMC11990772; doi:10.1371/journal.pcbi.1012948)
Supplement: S1 Text — (PDF) [file pcbi.1012948.s001.pdf]

Supplementary Materials

Integrating multiple spatial transcriptomics data using community-enhanced graph contrastive learning

Wenqian Tu and Lihua Zhang*

Content

SECTION 1 SUPPLEMENTARY FIGURES..1
SECTION 2 SUPPLEMENTARY TABLES 4

SECTION 1 SUPPLEMENTARY FIGURES

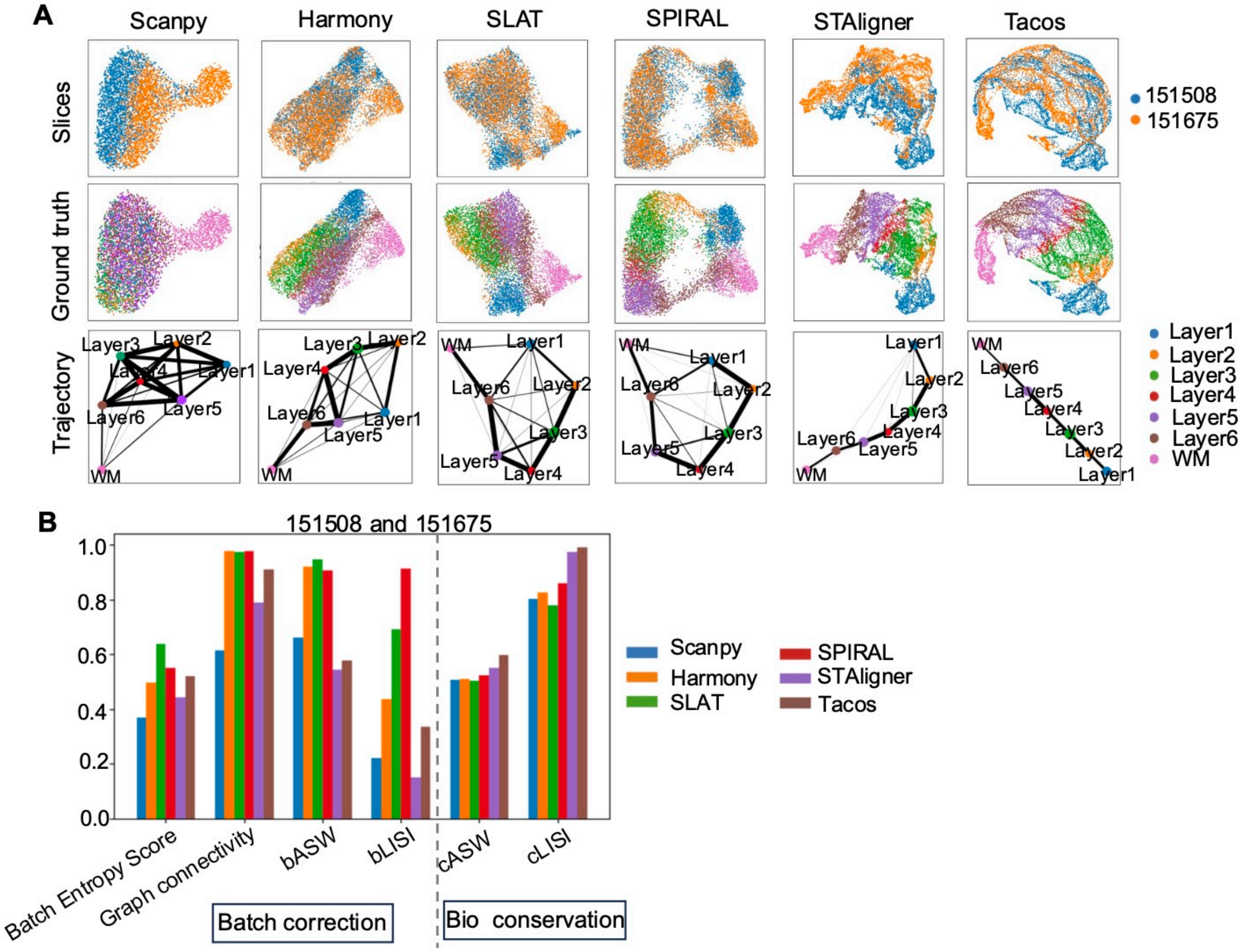

Fig. A. Benchmarking Tacos with other methods on slices 151508 and 151675 of DLPFC datasets from 10X Visium. (A) UMAP and PAGA visualization of aligned space of Scanpy, Harmony, SLAT, SPIRAL, STAligner and Tacos on slices 151508 and 151675. **(B)** Bar plots of different metric scores of aligned performances of these methods on slices of 151508 and 151675.

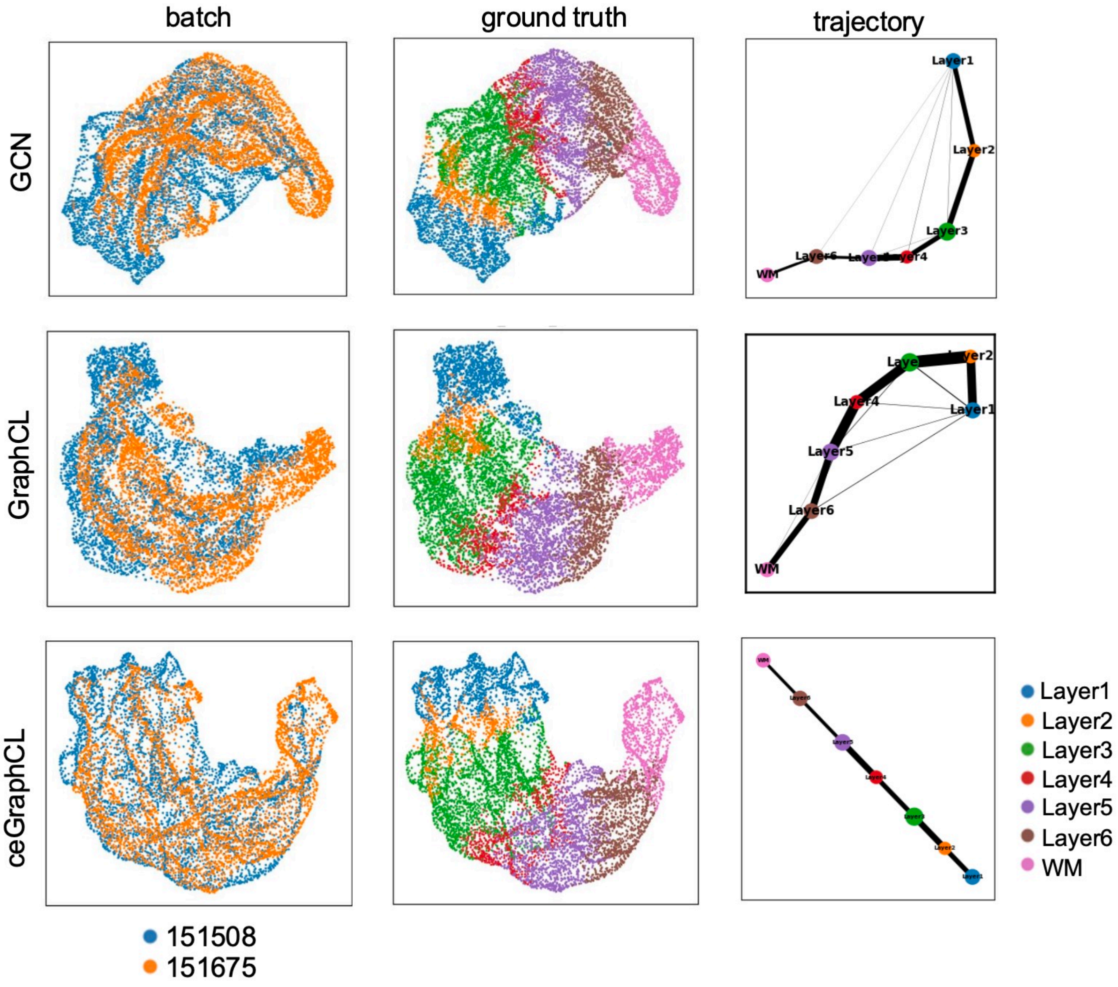

Fig. B. Comparison of the community-enhanced graph contrastive learning (ceGraphCL) in Tacos with GCN encoder and graph contrastive learning (GraphCL) on 151508 and151675 slices of DLPFC datasets. We replaced the community-enhanced graph contrastive learning with GCN encoder and GraphCL, while maintaining other structures in Tacos. UMAP and PAGA visualization (left) of aligned space of each method. On the UMAP, spots are colored by slices (left) and annotations (middle).

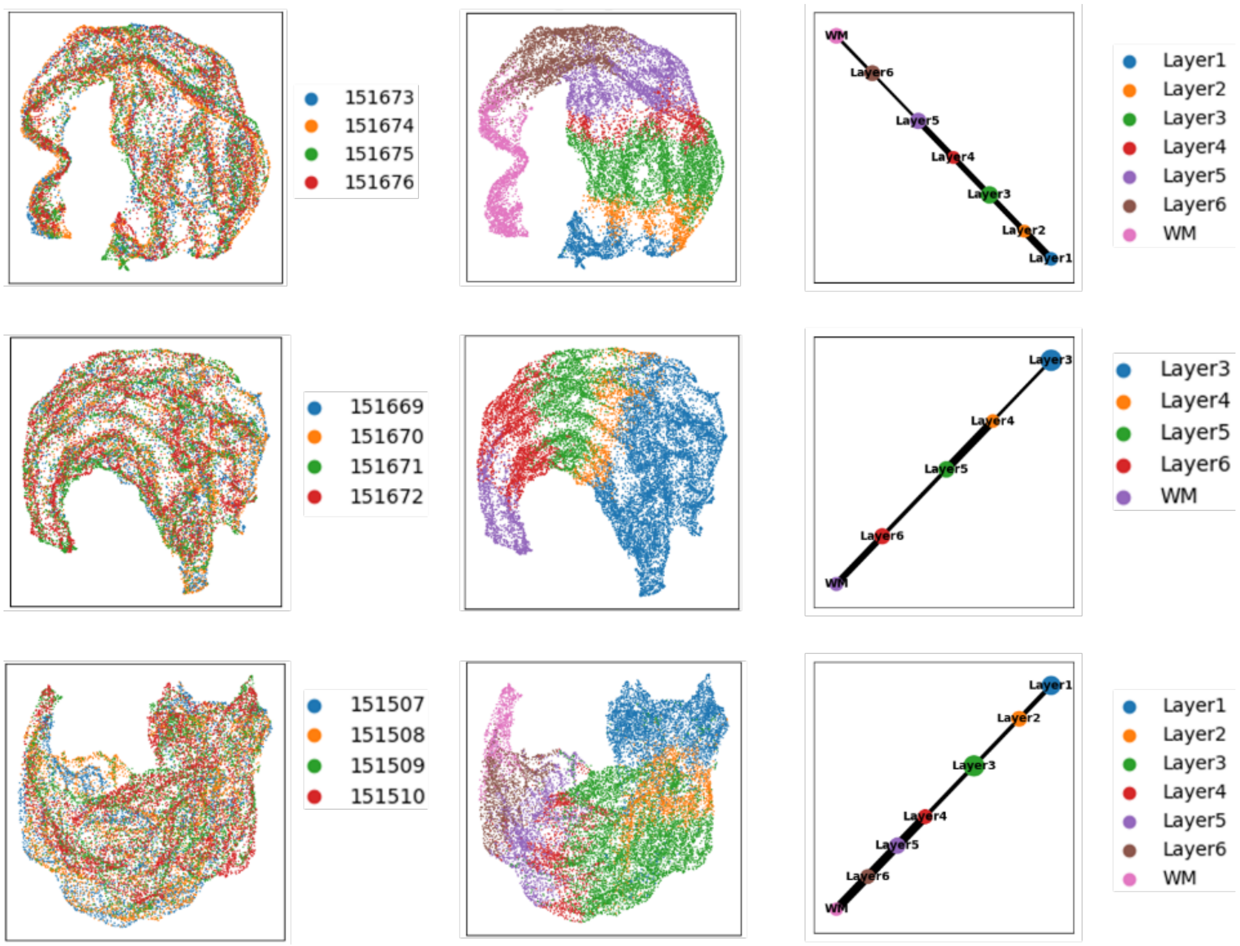

Fig. C. The performance of Tacos in integrating multi-slices of DLPFC dataset. UMAP and PAGA visualization (left) of aligned space of Tacos. On the UMAP, spots are colored by slices (left) and annotations (middle).

SECTION 1 SUPPLEMENTARY TABLES

Table A: Metric score of different graph-based methods on aligning DLPFC dataset.

Metric	151508 and 151675		
	GCN	GraphCL	ceGraphCL
Batch entropy score	0.592	0.618	**0.623**
Graph connectivity	1.0	1.0	**1.0**
bLISI	0.190	0.189	**0.190**
bASW	0.913	**0.947**	0.910
cASW	0.559	0.537	**0.574**
cLISI	0.984	0.962	**0.987**

Table B: Evaluation of the computational resources required for the datasets

Datasets	scales	FLOPs	parameters	training time (s/10 epochs)	memory (MB)
151508 and 151675 (DLPFC)	7924×4846	1,191,750	1,191,750	0.98	2,700
mouse embryo	14681×317	2,202,150	296,387	1.26	3,912
mouse olfactory bulb	32263×3236	1,191,750	1,191,750	3.15	14,252
mouse hippocampus	53058×4316	7,958,700	38,162,918	6.14	26,027
human brain (Xenium)	39427×264	5,914,050	224,042	4.02	21,519
