# Supplementary material for: Integrating multiple spatial transcriptomics data using community-enhanced graph contrastive learning
Source: PLoS Comput Biol. 2025 Apr 3;21(4):e1012948. doi: 10.1371/journal.pcbi.1012948 (PMC11990772; doi:10.1371/journal.pcbi.1012948)
Supplement: Attachment — Submitted filename: response_letterR1.pdf [file pcbi.1012948.s003.pdf]

# Response Letter for "Integrating multiple spatial transcriptomics data using community-enhanced graph contrastive learning"

Reviewer #1: Tu et al., in their research article titled "Integrating multiple spatial transcriptomics data using community-enhanced graph contrastive learning," showcase the use of graph learning method to integrate multiple spatial transcriptomics data from different platforms with different scenarios. The method development contributes to easing the complexities posed by spatial transcriptome data, especially the batch effect and noise impact. At its current stage, the article addresses technical defects of spatial transcriptomics rather than providing a certain degree of biological insights and aspects. The tool presented in the article is limited to integrating datasets from the same or different platforms, making the article in its present form unsuitable for publication. However, the article could be significantly improved if the authors added points as discussed below:

1) In the Overview of Tacos sub-section of the Result section, the author showcases a figure representing the schema of the graph theory algorithm. However, it is not mentioned why graph contrastive learning is the better choice. What were the criteria for selecting this algorithm? A better alternative will be to train different algorithms and finalize the algorithm that is best suited for the dataset. This validates and gives a better rationale for the tool development

**Response:** We apologize for the confusion. In the revised manuscript, we clarified this point. Graph neural network has been successfully adopted to deal with spatial transcriptomics data (*Hu et.al, Nat Methods, 2021; Li et.al., Nat Comput Sci, 2022; Dong et.al, Nat Commun, 2022; Long et.al, Nat Commun, 2023).* As the annotation information of the spatial transcriptomics data is usually unavailable, unsupervised learning methods are adopted to model the spatial transcriptomics data. Graph autoencoder and graph contrastive learning (GraphCL) are two common unsupervised graph neural networks. While the commonly used GraphCL builds augmented graphs by randomly mask edges or nodes. It treats each spot from a slice or across slices equally without considering the heterogeneously spatial structures within a tissue and without considering the different resolutions between different platforms, which may lead to limited performance in analyzing spatial transcriptomics data. Moreover, we compared the community-enhanced graph contrastive learning with these two types methods on slices 151508 and 151675 of DLPFC data. Specially, we replaced the community-enhanced graph contrastive learning with GCN encoder and GraphCL, while maintaining other structures in Tacos. We quantitively compared the alignment performance of these methods with six metrics. The community-enhanced graph contrastive learning approach consistently exhibited the best overall performance (new Supplementary Table S1). We also applied PAGA to the embeddings of each method and found that the community-enhanced graph contrastive learning method could accurately capture the liner structures between different layers than that of GCN and GraphCL (new Supplementary Figure S2). Therefore, we select the community-enhanced graph contrastive learning method. In the revised manuscript, we have added the points to the Results section and Supplementary file.

2) The author shows the smoothness of integration but fails to justify how non-computational biologists or bioinformaticians could utilize the present tool. Could the tool in its present form be integrated into well-known spatial transcriptomics analysis? If so, how could they integrate it?

**Response:** Yes, our tool can be integrated into well-known spatial transcriptomic analysis packages for downstream analysis such as domain identification and marker genes' detection. Actually, we utilized AnnData as the data container during preprocessing and stored the results of Tacos in the AnnData layer, which is same to the popularly used package Scanpy (https://scanpy.readthedocs.io/en/stable/tutorials/index.html) and Squidpy (https://squidpy.readthedocs.io/en/stable/). Therefore, our tool can be directly interface with existing these spatial transcriptomics analysis tools. In the revised version, we have updated the code in the Git repository (https://github.com/0617spec/tacos) to include this step, facilitating further analysis.

3) The tool's computational efficiency is not extensively discussed. Moreover, if the tool has extensive memory utilization, a section or supplementary note should discuss how multithreading or multiprocessing could elevate process execution.

**Response:** Thanks for the valuable suggestion. In the revised manuscript, we have added the computational efficiency of our approach on each dataset (new Supplementary Table S2). Large memory utilization is a critical issue for graph-based methods. To reduce memory usage, we have optimized our code by modifying the training process. Instead of feeding all slices into the model simultaneously, we process them individually during training, which can decrease memory requirements. Additionally, a lightweight graph convolutional approach in the community-enhanced graph learning step will be explored in the future. In the revised manuscript, we have added this point to the Supplementary file and Discussion section.

4) Both scRNA-seq and spatial transcriptomics are used to find novel biological markers or insights. However, the present tool does not provide a better alternative to the existing tool.

**Response:** Thanks for pointing it out. We have updated the code in the Git repository to interface with existing Scanpy (https://scanpy.readthedocs.io/en/stable/tutorials/index.html) and Squidpy (https://squidpy.readthedocs.io/en/stable/) for downstream analysis.

4) The GitHub page appears to be well maintained; however, the readme and license file need to be updated

**Response:** Thanks for pointing it out. In the revised version, we have updated the readme and license file.

Reviewer #2: The manuscript introduces Tacos, a novel community-enhanced graph contrastive learning-based method designed to integrate multiple spatial transcriptomics datasets across varying platforms and conditions. The method offers a promising tool for

integrating spatial transcriptomics data, with potential applications in studying tissue architecture and disease progression. However several areas could benefit from further clarification to improve its overall impact and scientific rigor.

1. Given the align output is in a low-dimensional embedding space. Besides to the comparison to the Scanpy integration without batch effect removal, it is preferable to also compare it with Scanpy's batch-corrected integration methods like Harmony. Additionally, SPIRAL has the same design for spatial data integration across various experiments and technologies, SPIRAL also test the performance on DLPFC and MOA datasets. it would be beneficial to include SPIRAL into the comparisons as well.

**Response:** Thanks for the valuable suggestion. We compared Tacos with Harmony and SPIRAL on the datasets used in the manuscript. Harmony and SPIRAL aligned different slices together. However, they had less performance than Tacos in capturing biological structures. For example, Tacos accurately detected the linear structures of different layers on low embeddings than Harmony and SPIRAL on adjacent slices 151674 and 151675 and non-adjacent slices 151508 and 151675 (updated Figure 2A-B). Moreover, SPIRAL had the over-alignment problem on MOB dataset and it failed to capture subtle differences between slices from different platforms. In contrast, Tacos was able to identify the specific structures, AOB and AOBgr (domain 7 and domain 8) in the Slide-seqV2 slices (updated Figure 3A).

2. How spatial gene expression data is denoised? A detailed explanation of the approach used to denoise spatial gene expression data are required.

**Response:** We apologize for the confusion. The low-dimensional embedding *Z* is fed into a two-layer GCN decoder with parametric rectified linear unit as activation function to reconstruct the gene expression profile. The reconstructed gene expression profile is treated as the denoised gene expression. The detailed method can be found in the Methods section titled "Spatial Transcriptomics Data Denoising." In the revised manuscript, we have added the citation of the Methods.

3. Compared to ground truth annotations, Tacos had less scores in preserving annotated layers, Could you clarify how these scores are defined? Additionally, does this imply that Tacos may not effectively preserve the biological structures represented by the annotated layers?

**Response:** Thanks for the question. We adopted six metrics to evaluate the performance. And the six metrics can be categorized into two types. The first type focuses on the batch effect removal including the batch entropy score, graph connectivity, bASW, and bLISI. For all these four metrics, higher scores indicate better alignment between batches. The second type measures the preservation of biological structure and includes cASW (isolated label ASW) and cLISI. The mathematical definitions of each metric can be found in the "Evaluation Metrics" section of the Methods. The metrics were calculated on the UMAP space of embeddings. In the revised manuscript, we have updated this point. Based on the biological preserve metrics

(cASW and cLISI), our method achieves the best performance. While SLAT performs better on batch-related metrics, it fails to effectively preserve the biological structure.

4. The trajectory tree analysis appears unconventional. Could you clarify how the hierarchical organization of the dorsolateral prefrontal cortex tissue was interpreted?

**Response:** Thanks for the question. The PAGA trajectory graph estimates the connectivity of manifold partitions, providing an interpretable, graph-like map of the data manifold. This allows for a clear visualization of the global topology of the embedding. We adopted PAGA on the embeddings obtained Tacos and other methods to check whether the embeddings could capture the global topology. In a PAGA graph, thicker edges indicate stronger connections. In comparison methods, connections are observed between non-adjacent layers, which are incorrected. In contrast, the PAGA trajectory graph generated from the embedding of Tacos exhibits a linear structure, consistent with the hierarchical structure of different layers. In the revised manuscript, we have clarified this point.

5. All the test datasets in the manuscript appear to involve two-sample integration. It would be valuable to assess the performance of Tacos in integrating multiple samples simultaneously.

**Response:** Thanks for the valuable suggestion. Tacos is capable to integrate multiple slices simultaneously. We applied it to integrate adjacent four slices of each donor on DLPFC data. We found that Tacos could also accurately detect the linear structures of adjacent layers (new Supplementary Figure S3). In the revised manuscript, we have added these results to the supplementary file.

6. Although spatial domain is not the direct output of Tacos, it is important to provide a detailed description of how spatial domains were determined. Additionally, clarify the criteria and methods used to compare spatial domains across different approaches.

**Response:** We apologize for the confusion. We identified spatial domains with mclust or community-graph based Louvain or Leiden algorithm on the obtained embeddings of Tacos and other methods. Specially, we recommend to use mclust for data from the 10x Visium platform, while we suggest to use Leiden for other platforms. In the revised manuscript, we have added this point to the Methods section.

Reviewer #3: In this study, the authors present TACOS, an advanced method for spatial transcriptomics integration that combines graph convolutional neural networks (GCNs) with innovative data preprocessing and augmentation strategies. By constructing cell graphs that integrate spatial and gene expression information, the method provides a robust framework for capturing the intricate relationships within spatial transcriptomics datasets. Furthermore, TACOS leverages contrastive learning to enhance feature representations, enabling the alignment of spatial datasets with unprecedented accuracy. This approach not only achieves state-of-the-art performance in spatial data integration but also addresses key challenges in

preserving spatial context and biological relevance. Importantly, the authors have developed TACOS as a Python package, ensuring its accessibility and utility for the research community. This work represents a significant advancement in the field of spatial transcriptomics, offering both methodological innovations and practical tools for researchers. While I recommend the paper for publication, addressing the following relatively minor comments will improve the manuscript's clarity and presentation.

1. Figure 1 and Caption: The figure does not provide adequate details to explain the model's training process. Specifically, the manuscript does not clarify how the deep learning model generates the aligned dataset or what specific steps are taken to achieve this alignment. Moreover, the caption does not describe the training objectives, such as the loss functions used in the model, or explain their significance in aligning the datasets. Adding these details would greatly enhance the clarity and accessibility of this figure, ensuring readers fully understand the underlying methodology.

**Response:** Thanks for the valuable suggestion. In the revised manuscript, we have updated Figure 1 and added the details to explain the model’s training process.

2. Methods Section – "Datasets and Data Preprocessing": There is a typographical error in the term "pseud-count," which should be corrected to "pseudocount." Additionally, the manuscript does not adequately discuss the choice of the number of highly variable genes, a parameter that is crucial for the analysis. This choice can significantly affect the results, and explicitly presenting the criteria used to select this parameter is necessary for reproducibility and transparency. A brief justification for the chosen number would strengthen the methodological rigor of the study.

**Response:** Thanks for pointing these out. We have corrected the typographical mistakes in the term "pseud-count" to "pseudocount" in the manuscript. In this study, we used all genes if the spatial transcriptomics data has fewer than 3000 genes. Otherwise, we selected high variable genes using the function sc.pp.highly_variable_genes with flavor="seurat_v3" in Scanpy package and top 3000 high variable genes were selected. Finally, genes in the intersection set of high variable genes of all slices were used. In the revised manuscript, we have added these details.

3. Methods Section – Graph Construction and Embeddings: The subsections "Building Spatial Graph for Spatial Transcriptomic Data" and "Extracting Low-Dimensional Embeddings with Community-Enhanced Encoder" require additional clarity in explaining the equations and symbols introduced. For example, the meaning of symbols such as "s" and "x" in the "1-skeleton" equation is not explained, leaving their interpretation ambiguous. Similarly, the rationale for using "V(s)" instead of directly constructing a k-nearest neighbor (KNN) graph based on spatial distances is not adequately articulated. Providing a clear explanation of how these techniques contribute to solving the spatial integration task would strengthen the section.

Additionally, numbering the equations for easier reference and avoiding redundancy in their presentation would improve the overall organization and readability of the manuscript.

**Response:** We apologize for the confusion. In the revised manuscript, we have clarified the details and added the numbers for the equations. KNN relies on a fixed number of neighbors, which may not adapt for spatial data with varying spatial densities. $V(s)$ uses geometric structures to define neighborhoods. This makes it more suitable for spatial data with non-uniform densities. In the revised manuscript, we have updated the "Building Spatial Graph for Spatial Transcriptomic Data".

4. Model Architecture and Training Details: The manuscript omits critical information about the structure and optimization of the deep learning model. Details such as the architecture of each module during training, including the number of neurons, are not provided. Similarly, essential optimization parameters, such as the choice of optimizer, learning rate, and the number of training epochs, are missing. Furthermore, there is no discussion of runtime or computational efficiency, particularly concerning graph construction and graph convolutional operations. Since the method involves graph-based spot-level interactions, it is important to clarify whether full-batch mode is used for graph convolutions and, if so, discuss its implications for runtime and memory requirements. Including these details would provide a more comprehensive understanding of the computational aspects of the method.

**Response:** Thanks for the valuable suggestion. In the revised manuscript, we have added the details about the structure, training strategy and the computational efficiency in the Methods section and supplementary file.

5. Figure 2 and Baseline Comparisons: The use of Leiden clustering on embeddings derived from PCA in Scanpy is not appropriate. Leiden clustering is primarily designed for identifying cell types based on gene expression, not for analyzing spatial slices, making its relevance to this context questionable. Additionally, the comparison with the ground truth trajectory is unclear. The manuscript mentions performing PAGA using ground truth labels, but this approach does not seem to provide a fair baseline for evaluating spatial integration tasks. A more suitable baseline comparison that directly addresses the spatial integration objectives of the study would better validate the effectiveness of TACOS and bolster the credibility of the results.

**Response:** Thanks for the question. In the revised manuscript, we compared Tacos with spatial integration methods (SLAT, STAligner, SPIRAL) and non-spatial integration method (Harmony) as well as non-integration method (Scanpy). We comprehensively assessed the effectiveness of spatial integration with six different evaluation metrics. And the six metrics can be categorized into two types. The first type focuses on the batch effect removal including the batch entropy score, graph connectivity, bASW, and bLISI. For all these four metrics, higher scores indicate better alignment between batches. The second type measures the preservation of biological structure and includes cASW (isolated label ASW) and cLISI. We carefully check

the manuscript, we used the annotation layers in computing the metrics rather than Leiden clustering on this data to directly evaluate the performance. We adopted PAGA on the integrated embeddings of Tacos and other methods to check whether the integrated embeddings could capture the biological structures of the data. A good integrated embedding should preserve the linear structure of different layers. In the revised manuscript, we have clarified these points.
